# Study on the Optimization of FDM Parameters for the Manufacture of Three-Point Bending Specimens from PETG and Recycled PETG in the Context of the Transition to the Circular Economy

**DOI:** 10.3390/polym17121645

**Published:** 2025-06-13

**Authors:** Dragos Valentin Iacob, Dragos Gabriel Zisopol, Mihail Minescu

**Affiliations:** 1 Department of Mechanical Engineering, Doctoral School, Petroleum-Gas University of Ploiesti, 100680 Ploiesti, Romania; dragoshicb@gmail.com; 2 Mechanical Engineering Department, Petroleum-Gas University of Ploiesti, 100680 Ploiesti, Romania; mminescu@upg-ploiesti.ro

**Keywords:** bending, optimization, FDM parameters, experimental determinations, flexural stress

## Abstract

This study presents the results of optimizing FDM 3D printing parameters (layer height deposited in one pass—L_h_ and filling density—I_d_) to enhance the mechanical performance of three-point bending specimens made from PETG and recycled PETG (rPETG). The objectives of the study are to investigate the influence of variable parameters (L_h_ and I_d_) on the three-point bending behavior of additively manufactured specimens by thermoplastic extrusion of PETG and rPETG. It is also aims to optimize the manufacturing parameters to maximize mechanical performance, but also to evaluate the potential of using rPETG in mechanical engineering applications. The materials analyzed in this study are PETG and recycled PETG (rPETG), in the context of promoting the concept of circular economy. Using the QIDI Q1 Pro 3D printer, and the variable parameters of FDM, L_h_ = (0.10; 0.15; 0.20) mm and I_d_ = (50; 75; 100)%, 90 three-point bending specimens (45 from PETG and 45 from rPETG) were additively manufactured. To determine the mechanical strength characteristics under three-point bending stress, all 90 additively manufactured specimens were tested in three-point bending using a Barrus White 20 kN universal testing machine. The maximum bending stress is influenced by the two considered variable parameters of FDM (L_h_ and I_d_), the parameter with the greater impact being I_d_. Comparing the results of the maximum bending stresses of the additively manufactured specimens made of PETG and rPETG using the optimal parameters, it was found that the maximum bending stresses are higher in the case of the rPETG specimens, which highlights the potential of using recycled plastics in mechanical engineering applications.

## 1. Introduction

Given the current economic context, the climate crisis and resource depletion, the transition to a circular economy has become a major global priority [1,2,3,4,5,6,7]. The manufacturing industry is forced to find opportunities to reduce production costs, reduce waste and make production processes more flexible [8,9,10,11,12]. Additive manufacturing technologies, especially fused deposition modeling (FDM), offer significant advantages over conventional manufacturing technologies (formative and subtractive) in terms of material efficiency, the ability to create complex geometries and sustainability. In contrast to subtractive processes which generate significant material losses leading to low material utilization indexes or to formative processes, additive manufacturing technologies stand out due to the principle of functionality, which is based on the successive addition of material layer. The proposed approach of additive manufacturing technologies minimizes the amount of waste resulting from the production process [13,14,15,16,17,18,19,20,21,22,23,24,25]. FDM is one of the most popular additive manufacturing technologies due to its accessibility, and is also a technology that can easily adopt the use of recycled plastics [26,27,28,29,30,31,32,33,34,35,36]. One of the main challenges in adopting recycled plastics is the use of a large amount of recycled material without affecting mechanical performance. PETG (polyethylene terephthalate glycol) is a thermoplastic material widely used for various applications such as packaging, medical and pharmaceutical device manufacturing, 3D printing, industrial and engineering applications [11,14,25]. Printing parameters significantly influence the mechanical properties and manufacturing costs of parts [10,11,26,28]. Optimization of parameters is essential in the context of efficient use of resources, and is highlighted in the following specialized works. In the paper [37], the influence of FDM parameters (filling density, number of wall perimeters, layer height deposited in one pass) on the mechanical characteristics of parts manufactured by thermoplastic extrusion of PLA is studied. The results of the study show that by increasing the filling density and the number of wall perimeters and by decreasing the layer height deposited in one pass, increases in the rigidity of the parts are obtained. In the paper [38], the authors investigate the influence of filling density and printing speed on the breaking strength of tensile specimens additively manufactured from PLA. Artificial neural networks (ANN) trained on experimental data were used to predict the mechanical behavior of tensile specimens. The conclusions of the study show that increasing the filling density results in an increase in the tensile strength of the specimens, and increasing the printing speed from 5 mm/s to 35 mm/s resulted in a decrease in the tensile strength. The research presented in the paper [39] analyzes the influence of the filling pattern and filling density on the mechanical properties of PLA/Cu composites 3D printed by FDM. The results of the study show that the highest results of the tensile strength were recorded for the parts manufactured with a filling density of 90% and the linear filling pattern. In the paper [40], the authors present the results of the study on the optimization of FDM parameters to maximize the flexural strength. For the study, ABS three-point bending test specimens were additively fabricated according to ASTM D790 using the following FDM parameters: layer height, L_h_ = (0.10; 0.20; 0.30) mm; extrusion temperature, E_t_ = (220; 230; 240) °C; and printing speed, P_s_ = (30; 50; 70) mm/s. The study conclusions show that the highest flexural strength (56.65 MPa) was obtained for the additively fabricated specimen using the following parameters: L_h_ = 0.10 mm; E_t_ = 230 °C; and P_s_ = 30 mm/s. Before using recycled materials in practical applications, it is necessary to carry out a rigorous evaluation of the behavior under specific mechanical stresses such as tensile, compression, three-point bending, and resilience. In the paper [41], the study on the tensile behavior of specimens manufactured by FDM from rPETG is presented; the research results show that the tensile strength of specimens manufactured from recycled material is higher by up to 31.59% compared to the tensile strength of specimens manufactured from virgin PETG. In the paper [42], the study on the optimization of FDM parameters for the manufacture of compression specimens from rPETG is presented; the conclusions of the study show that the compressive strengths of specimens manufactured from rPETG are higher by 11.39–25.91% compared to the compressive strengths of specimens manufactured from virgin PETG. In [43], the authors present a study on the valorization of HDPE waste and industrial glass fibers by integrating additive manufacturing technologies into applications through plastic extrusion. The results of the study show that the composite mixture formed by HDPE waste and glass fibers offers good mechanical characteristics; this material mix can be used for the manufacture of functional parts.

The article [44] provides an analysis of the progress of using recycled PET in applications of additive manufacturing technologies through plastic extrusion. PET waste is mostly derived from packaging that requires rigorous processing consisting of washing, drying, grinding and extruding the material into filament. The technical challenges identified are represented by residual moisture, thermal degradation, but also by low viscosity. The solutions identified to mitigate the technical problems are represented by compatibilization with other polymers, the addition of stabilizers and the optimization of process parameters. The study presented in [45] investigates the potential of composites obtained from industrial waste polypropylene with glass fibers, which are first processed by 3D printing, and subsequently recycled and reprocessed by injection molding in order to obtain new components. The results of the study demonstrate the viability of reusing composite waste using a hybrid method (3D printing and injection molding). The results of the study presented in [46] demonstrate the viability of using composites made of PETG with short carbon fibers and which are subjected to heat treatment, which have excellent applicability for the production of functional parts due to their high mechanical performance.

This work is part of a larger research study that examines the influence of variable FDM parameters (L_h_ and I_d_) on the mechanical behavior of additively manufactured parts made of PETG and rPETG. In previous research, the authors studied other types of mechanical tests, such as tensile and compression tests [41,42].

In this paper, the influence of variable FDM parameters (L_h_—layer height deposited in one pass and I_d_—filling density) on the mechanical performance of PETG and rPETG specimens in three-point bending is studied. Using statistical analysis and optimization methods, the optimal parameters for the manufacture of three-point bending specimens were identified. This paper serves as a basis for further research on the integration of recycled materials in the field of additive manufacturing technologies through plastic extrusion; at the same time, this paper brings value to the specialized literature, filling existing gaps.

## 2. Materials and Methods

### 2.1. Manufacturing of Specimens for Three-Point Bending Test

**Figure 1:**
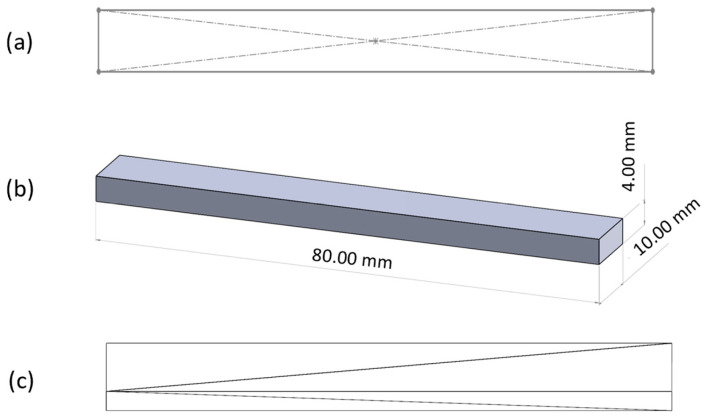
Three-point bending test specimen in Solidworks 2023: (**a**) 2D sketch; (**b**) 3D model; (**c**) STL model.

The 2D sketch (Figure 1a) and subsequently the 3D model (Figure 1b) of the three-point bending specimen, according to the ISO 178:2019 standard, was created using the Solidworks 2023 CAD software [47]. The 3D model corresponding to the three-point bending specimen (Figure 1c) was converted from SLD format to STL format using the same CAD software.

**Figure 2:**
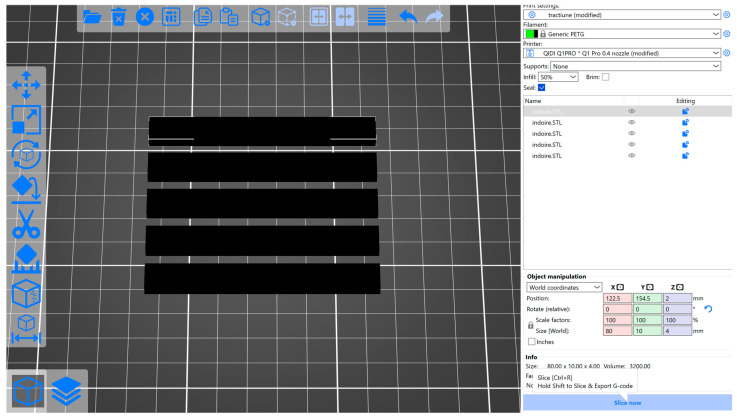
Specimens for three-point bending test in QIDISlicer [48].

The STL file corresponding to the specimen for the three-point bending test was processed in the QIDISlicer software version 1.2.1, (see Figure 2), where the process parameters were set according to Table 1 and the work instructions in G-Code format were generated.

**Figure 3:**
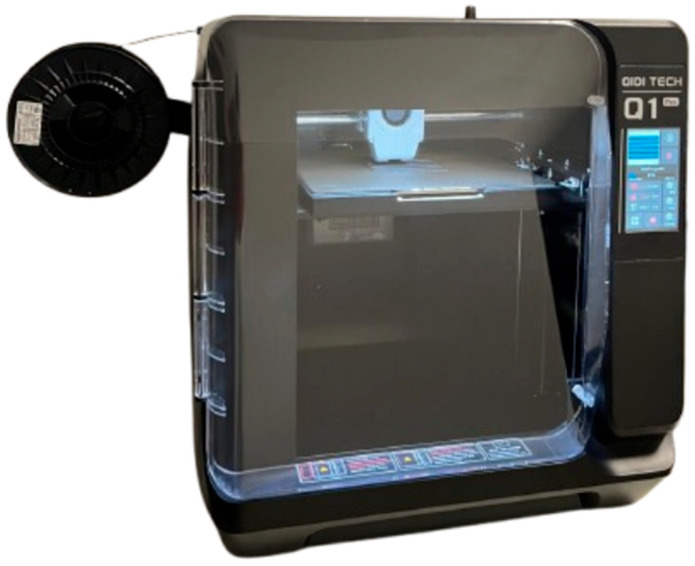
Three-dimensional printer—QIDI Q1 Pro, used to manufacture three-point bending specimens from PETG and rPETG.

The G-Code files were transferred to the QIDI Q1 Pro 3D printer (WenZhou, China), where 90 three-point bending specimens (45 made of PETG and 45 made of rPETG) were additively manufactured by thermoplastic extrusion. To manufacture the parts, 1.75 mm diameter filament made of PETG and rPETG with 100% recycled material from the Everfil brand (Białystok, Poland) was used, (see Figure 3).

**Figure 4:**
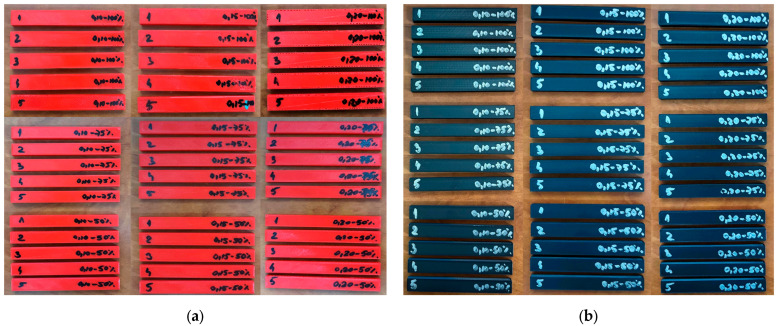
Three-point bend test specimens manufactured on the QIDI Q1 Pro printer: (**a**) PETG; (**b**) rPETG.

Figure 4 shows the 90 three-point bending test specimens manufactured on the QIDI Q1 Pro 3D printer.

### 2.2. Three-Point Bending Test of Additively Manufactured PETG and rPETG Specimens

**Figure 5:**
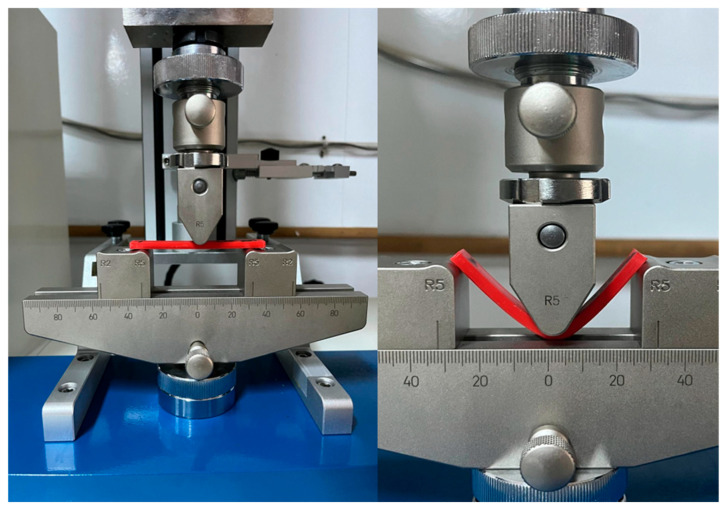
Three-point bending test on the Barrus White 20 kN universal testing machine.

All 90 specimens (45 PETG and 45 rPETG) were tested in three-point bending on the Barrus White 20 kN universal testing machine (Budapest, Hungary), according to ISO 178:2019 standard, using a speed of 5 mm/min, (see Figure 5) [47].

**Figure 6:**
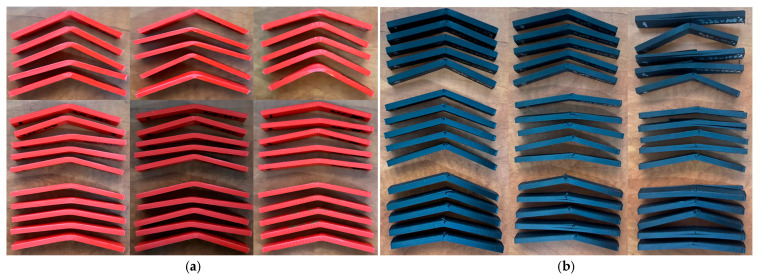
Specimens manufactured on the QIDI Q1 Pro printer after three-point bending test: (**a**) PETG; (**b**) rPETG.

Figure 6 shows the 90 specimens after the three-point bending test on the Barrus White 20 kN universal testing machine.

## 3. Results and Discussion

**Figure 7:**
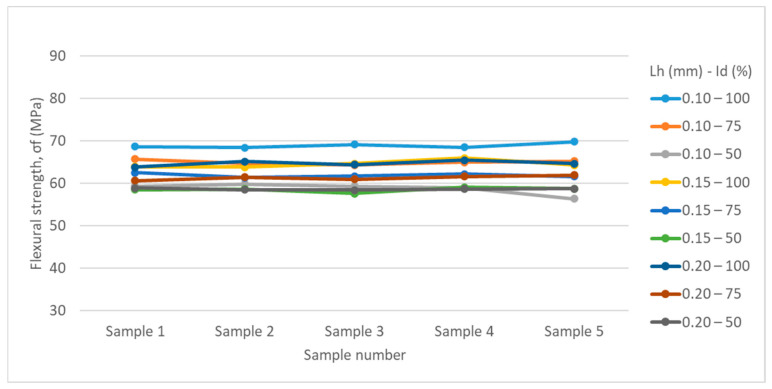
Graphical representation of the three-point flexural strengths of specimens made of PETG.

**Figure 8:**
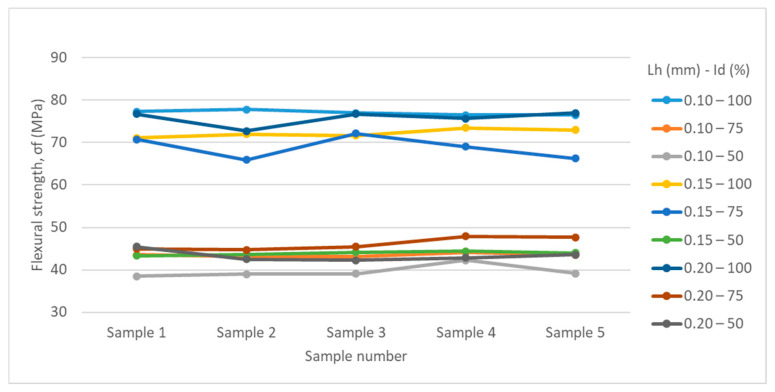
Graphical representation of the three-point flexural strengths of specimens manufactured from rPETG.

Table 2 and Table 3 and Figure 7 and Figure 8 summarize the results obtained from the experimental determinations of three-point bending of PETG and rPETG specimens on the Barrus White 20 kN universal testing machine.

Analyzing the data in Table 2 and Figure 7, we observe that the maximum flexural strength (69.78 MPa) was obtained for specimen 5 from the set manufactured with parameters L_h_ = 0.10 mm and I_d_ = 100%, and the minimum flexural strength (56.37 MPa) was obtained for specimen 5 from the set manufactured with L_h_ = 0.10 mm and I_d_ = 50%. Analyzing the average values of the flexural strengths, we observe that the maximum value (68.87 MPa) was obtained for the specimens manufactured with L_h_ = 0.10 mm and I_d_ = 100%, and the minimum value of the flexural strengths (58.49) was obtained for the specimens manufactured with L_h_ = 0.15 mm and I_d_ = 50%.

Reducing the filling density (I_d_) from 100% to 75% led to a decrease in flexural strengths of 4.96–5.71%, and by reducing the filling density from 75% to 50%, the flexural strengths decreased by 4.57–9.53%.

Analyzing the data presented in Table 3 and Figure 8, we observe that the maximum flexural strength (77.01 MPa) was obtained for specimen 2 from the set manufactured with parameters L_h_ = 0.10 mm and I_d_ = 100%, and the minimum flexural strength (38.48 MPa) was obtained for specimen 1 from the set manufactured with L_h_ = 0.10 mm and I_d_ = 50%. Analyzing the average values of the flexural strengths, we observe that the maximum value (77.01 MPa) was obtained for the specimens manufactured with L_h_ = 0.10 mm and I_d_ = 100%, and the minimum value of the flexural strengths (39.59) was obtained for the specimens manufactured with L_h_ = 0.10 mm and I_d_ = 50%.

Reducing the filling density (I_d_) from 100% to 75% led to a decrease in flexural strengths by 10.61–39.79%, and by reducing the filling density from 75% to 50%, the flexural strengths decreased by 8.98–36.27%.

Analyzing Figure 8, we observe that the flexural strengths of rPETG specimens are considerably affected by the reduction in filling density.

**Figure 9:**
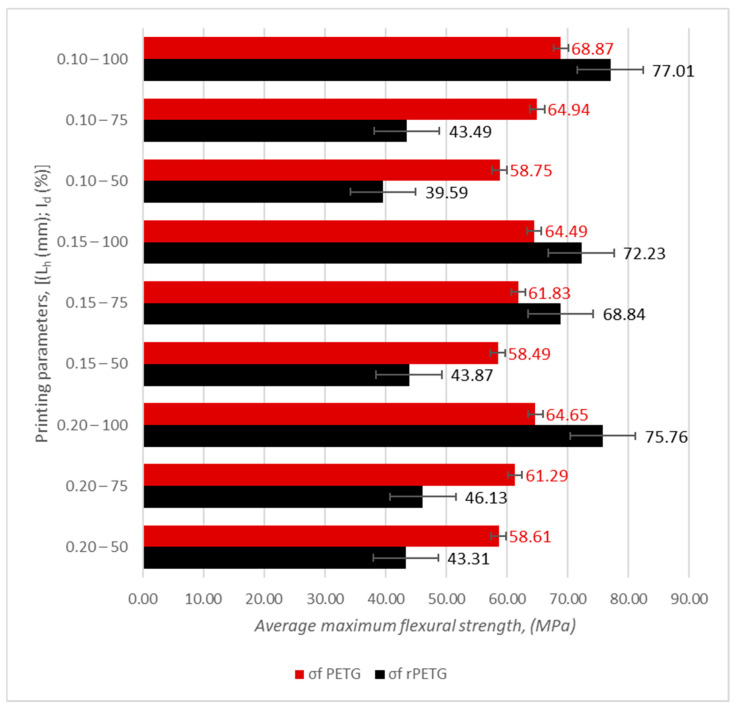
Average values of flexural strengths of PETG and rPETG specimens.

Figure 9 shows a comparison of the average values of the three-point flexural strength for specimens manufactured by thermoplastic extrusion of PETG and rPETG.

Analyzing Figure 9, we observe that the variable printing parameters (L_h_ and I_d_) and the material (PETG and rPETG) significantly influence the flexural strengths of the samples additively manufactured on the QIDI Q1 Pro 3D printer.

The flexural strength results obtained for the samples manufactured from rPETG with I_d_ = 100% are higher by 10.58–10.72% compared to the flexural strengths results corresponding to the samples manufactured from PETG using the same parameters.

The flexural strengths values obtained for the samples manufactured from rPETG with I_d_ = 75% are higher up to 6.02% compared to the flexural strengths results of the samples manufactured from PETG using the same parameters.

The flexural strengths of the specimens made of rPETG with I_d_ = 50% are lower by 25.32–32.32% compared to the flexural strengths of the specimens made of PETG using the same parameters.

The average of all values of the flexural strengths of the specimens made of PETG with L_h_ = (0.10; 0.15; 0.20) mm and I_d_ = (50; 75; 100)% is higher by 9.19% compared to the average flexural strengths of the specimens made of rPETG.

The superior performance of the samples manufactured from rPETG with I_d_ = 100% is the result of improved adhesion of the overlapping layers, but also of increased crystallinity.

**Figure 10:**
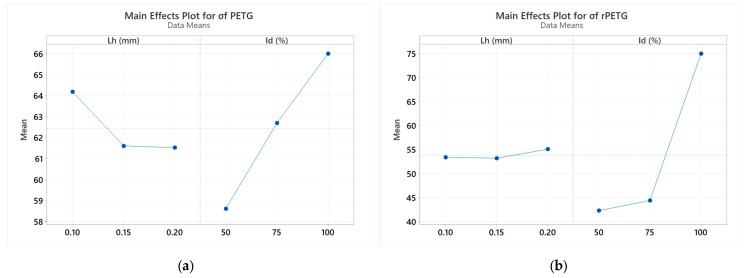
Effect diagrams of factors influencing flexural strength: (**a**) PETG; (**b**) rPETG.

To evaluate the influence of the FDM process parameters, namely layer height (Lh) and infill density (Id) on the three-point flexural strengths of additively manufactured PETG and rPETG specimens, an ANOVA (Analysis of Variance) analysis was performed using Minitab 19 software [49]. The results obtained from this statistical analysis are presented in Figure 10. The *p*-value plays a crucial role in determining the statistical significance of the influence of factors on an analyzed characteristic. For PETG, the corresponding *p*-values for L_h_ and I_d_ are 0.987 and 0.0051, respectively. In the case of rPETG, the obtained *p*-values for L_h_ and I_d_ are 0.591 and 0.0001, respectively.

The graphs presented in Figure 10 show how the variable parameters of thermoplastic extrusion (L_h_ and I_d_) influence the mechanical performance of PETG and rPETG specimens in three-point bending. Interpreting the data in Figure 10a, we find that the filling density (I_d_) is the parameter that significantly influences the bending resistance of PETG additively manufactured specimens. At the same time, a decrease in bending resistance is observed due to the increase in the height of the deposited layer at a transition from 0.10 to 0.15 and 0.20 mm, respectively. Analyzing Figure 10b, we observe the major impact of the filling density on the bending resistance.

**Figure 11:**
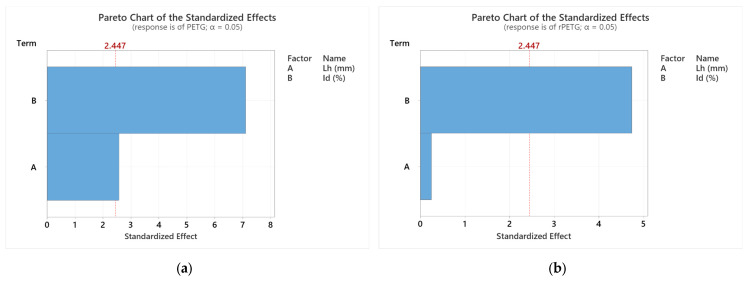
Pareto charts regarding the influence of variable parameters A = L_h_ and B = I_d_ on flexural strengths: (**a**) PETG; (**b**) rPETG.

Figure 11 presents Pareto graphs regarding the influence of variable thermoplastic extrusion parameters on the flexural strengths of specimens made of PETG and rPETG.

Analyzing Figure 11a, we observe that both parameters (A = L_h_ and B = I_d_) influence the flexural strengths of PETG specimens, the factor B = I_d_ having a 63.85% greater influence than the factor with A = L_h_. Evaluating the graph in Figure 11b, we observe the decisive influence of the factor B = I_d_ on the flexural strengths of specimens manufactured from rPETG. The influence of the factor A = L_h_ is insignificant, being 94.79% lower than the influence of the factor B = I_d_.

**Figure 12:**
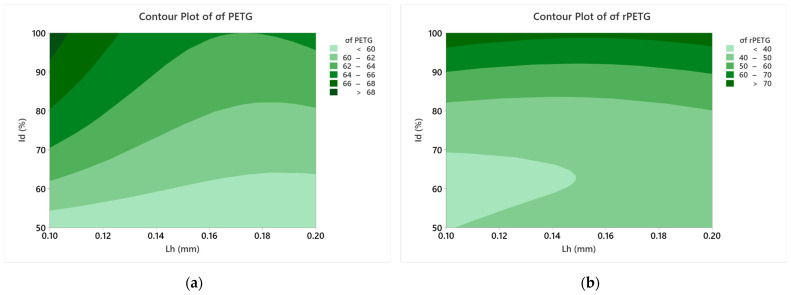
Contour plots corresponding to the influence of Lh and Id on the flexural strengths: (**a**) PETG; (**b**) rPETG.

Figure 12 presents the contour plots regarding the influence of the parameters L_h_ and I_d_ on the three-point flexural strengths.

Analyzing Figure 12, we observe how L_h_ and I_d_ influence the flexural strengths of specimens manufactured from PETG and rPETG. The graph in Figure 12a shows the strong influence of I_d_ and the moderate influence of L_h_; by increasing the filling density (I_d_), higher values of flexural strengths are obtained. The graph shown in Figure 12b shows that I_d_ is the critical factor for the flexural strength of specimens manufactured additively from rPETG. By increasing the filling density, a clear increase in flexural strengths is obtained.

**Figure 13:**
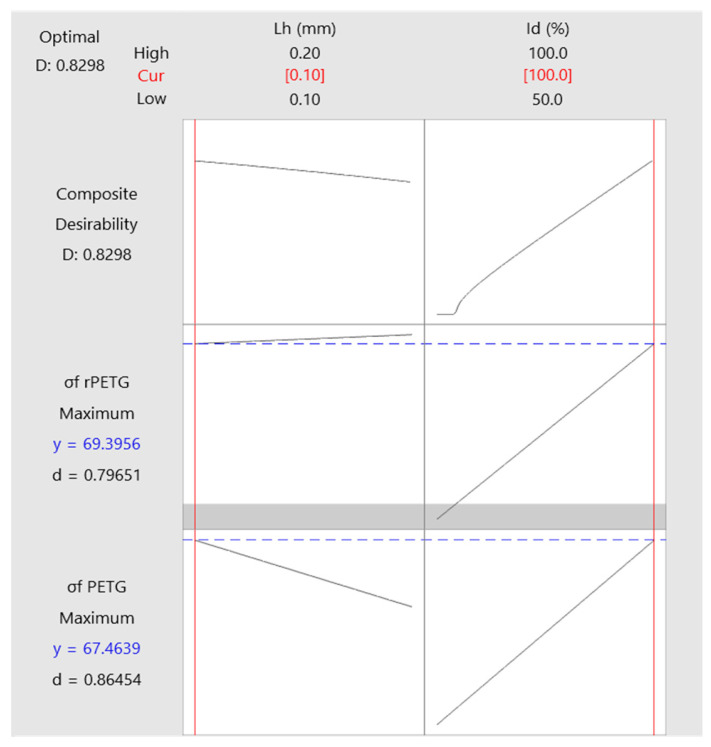
Manufacturing parameter optimization plots for PETG and rPETG three-point bending specimens.

Figure 13 shows the optimization plots of the variable manufacturing parameters (L_h_—deposited layer height and I_d_—filling density) for specimens made of PETG and rPETG, in order to maximize the three-point coving resistance. The optimization results show that for both materials, the optimal configuration for maximizing the three-point flexural strength is L_h_ = 0.10 mm and I_d_ = 100%.

Using Minitab and the variable parameters of thermoplastic extrusion in Table 1, regression equations were obtained with which the three-point bending strength can be predicted for each type of material studied.

(1) $$\sigma f\;PETG=55.36-26.7\;Lh\;\left(mm\right)+0.1477\;Id\;(\%)$$

(2) $$\sigma f\;rPETG=2.2+17.0\;Lh\;\left(mm\right)+0.655\;Id\;(\%)$$

## 4. Conclusions

This paper presents the results of research on optimizing FDM parameters for the manufacture of PETG and recycled PETG bending specimens in the context of the transition to a circular economy. For the study, 45 three-point bending specimens of PETG and 45 three-point bending specimens of rPETG were manufactured on the QIDI Q1 Pro 3D printer, which were subsequently tested on the Barrus White 20 kN universal testing machine.

- Printing parameters (layer height L_h_ and infill density I_d_) significantly influence the bending strengths of PETG and rPETG specimens (see Figure 10, Figure 11 and Figure 12).
- Optimal parameters for maximum flexural strength:
- L_h_ = 0.10 mm and I_d_ = 100%.

Key findings:

- rPETG outperforms PETG under optimal conditions, showing 10.72% higher bending resistance.
- The average bending resistances of the 45 PETG specimens are 9.19% higher compared to the average bending resistances of rPETG specimens.

Statistical validation:

- Infill density (I_d_) have the most influence on mechanical performance, (see Figure 10, Figure 11 and Figure 12).

The results obtained in this study highlight the potential of using recycled materials in mechanical engineering applications for the manufacture of functional parts that are subject to moderate loads. The use of recycled materials helps to reduce the negative impact on the environment caused by the amount of plastic waste. Another benefit of using recycled materials in applications of additive manufacturing technologies through plastic extrusion is the reduction in production costs, the costs of recycled materials being lower compared to those of virgin materials.

Given the contrast between the results obtained in this study and the superior mechanical results obtained for rPETG in the works [41,42], the authors propose to study the internal structures of the parts, the effect of recycling on material adhesion, and the bonding between the layers in our future research.

The authors propose to extend this research by carrying out a technical–economic study based on maximizing the ratio between the use value (flexural strength) and the production cost. At the same time, the authors propose to extrapolate this study to other types of materials.

## Figures and Tables

**Table 1:** FDM printing parameters used to manufacture flexural samples from PETG and rPETG.

Printing Parameters	PETG; rPETG
Part orientation, P_o_	X-Y
Extruder temperature, E_t_	250 °C
Platform temperature, P_t_	70 °C
Printing speed, P_s_	120 mm/s
Plate adhesion, P_a_	Brim
Layer height, L_h_	0.10; 0.15; 0.20 mm
First layer height, F_lh_	0.30 mm
Top solid layers, T_l_	5
Bottom solid layers, B_l_	3
Infill density, I_d_	50; 75; 100%
Infill pattern, I_p_	Rectilinear
Fill angle, F_a_	45°
Top fill pattern, T_f_	Monotonic lines
Bottom fill pattern, B_f_	Monotonic lines
Extrusion multiplier, E_m_	0.95
Pressure advance, P_a_	0.086 mm/s
Chamber fan speed, C_fs_	100%
Nozzle diameter, N_d_	0.40 mm

**Table 2:** Results of three-point bending tests of specimens made of PETG.

Layer Height, L_h_	Infill Density, I_d_	Maximum Flexural Strength, σ_f_					Average
**(mm)**	**(%)**	**Sample Number**					**(MPa)**
		**1**	**2**	**3**	**4**	**5**	
0.10	100%	68.61	68.37	69.08	68.49	69.78	68.87
	75%	65.67	64.61	64.25	64.96	65.19	64.94
	50%	59.31	59.78	59.31	58.96	56.37	58.75
0.15	100%	63.90	63.78	64.61	65.90	64.25	64.49
	75%	62.49	61.31	61.67	62.14	61.55	61.83
	50%	58.49	58.61	57.55	59.08	58.72	58.49
0.20	100%	63.78	65.08	64.37	65.43	64.61	64.65
	75%	60.61	61.43	60.96	61.55	61.90	61.29
	50%	58.84	58.49	58.37	58.61	58.72	58.61

**Table 3:** Results of three-point bending tests of specimens manufactured from rPETG.

Layer Height, L_h_	Infill Density, I_d_	Maximum Flexural Strength, σ_f_					Average σ_f_
**(mm)**	**(%)**	**Sample Number**					**(MPa)**
		**1**	**2**	**3**	**4**	**5**	
0.10	100%	77.32	77.79	76.96	76.49	76.49	77.01
	75%	43.54	43.19	43.07	44.13	43.54	43.49
	50%	38.48	38.95	39.07	42.25	39.19	39.59
0.15	100%	71.08	72.02	71.67	73.43	72.96	72.23
	75%	70.81	65.90	72.18	69.04	66.29	68.84
	50%	43.31	43.54	44.13	44.37	44.01	43.87
0.20	100%	76.73	72.73	76.73	75.67	76.96	75.76
	75%	44.95	44.72	45.42	47.90	47.66	46.13
	50%	45.42	42.48	42.25	42.84	43.54	43.31

## Data Availability

Data are contained within the article.
